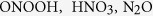# Aqueous reactive species induced by a surface air discharge: Heterogeneous mass transfer and liquid chemistry pathways

**DOI:** 10.1038/srep23737

**Published:** 2016-04-01

**Authors:** D. X. Liu, Z. C. Liu, C. Chen, A. J. Yang, D. Li, M. Z. Rong, H. L. Chen, M. G. Kong

**Affiliations:** 1 State Key Lab of Electrical Insulation and Power Equipment, Center for Plasma Biomedicine, Xi’an Jiaotong University, Shaanxi, P R China; 2 Frank Reidy Center for Bioelectrics, Old Dominion University, Norfolk, Virginia 23508, USA; 3 Department of Electrical and Computer Engineering, Old Dominion University, Norfolk, Virginia 23529, USA

## Abstract

Plasma-liquid interaction is a critical area of plasma science and a knowledge bottleneck for many promising applications. In this paper, the interaction between a surface air discharge and its downstream sample of deionized water is studied with a system-level computational model, which has previously reached good agreement with experimental results. Our computational results reveal that the plasma-induced aqueous species are mainly H^+^, nitrate, nitrite, H_2_O_2_ and O_3_. In addition, various short-lived aqueous species are also induced, regardless whether they are generated in the gas phase first. The production/loss pathways for aqueous species are quantified for an air gap width ranging from 0.1 to 2 cm, of which heterogeneous mass transfer and liquid chemistry are found to play a dominant role. The short-lived reactive oxygen species (ROS) and reactive nitrogen species (RNS) are strongly coupled in liquid-phase reactions: NO_3_ is an important precursor for short-lived ROS, and in turn OH, O_2_^−^ and HO_2_ play a crucial role for the production of short-lived RNS. Also, heterogeneous mass transfer depends strongly on the air gap width, resulting in two distinct scenarios separated by a critical air gap of 0.5 cm. The liquid chemistry is significantly different in these two scenarios.

## Introduction

Cold atmospheric-pressure plasmas have great prospects in various application fields such as biomedicine, wastewater treatment, agriculture and nano-technology^1^,^2^,^3^,^4^. Although the plasma-generated reactive species especially reactive oxygen species (ROS) and reactive nitrogen species (RNS) are widely thought to play a dominant role in many applications, the knowledge of such species is mostly limited in the gas phase, not directly relevant to the targets to be treated in a moisture environment or in bulk liquids. Given that some gaseous species are capable of penetrating only very short distances into water, ~2.5 nm in the case of electrons for instance, plasma-induced aqueous species which can directly act on targets are very different from the gas phase^5^,^6^,^7^,^8^. At present, how gaseous reactive species may be correlated to their aqueous counterparts is far from well understood, nor are physicochemical behaviors of reactive species in plasma activated water (PAW)^5^,^6^,^7^,^8^,^9^,^10^. Plasma-liquid interaction is a critical area of plasma science and a knowledge bottleneck for many promising applications.

In this paper, the interaction between a surface air discharge and the downstream dish of deionized water is studied. Our aim is to quantify the density profiles of the aqueous reactive species, and to map their production pathways. Surface air plasmas have recently been used in a number of important applications^11^,^12^,^13^,^14^,^15^, and their electrical disconnection from a downstream sample allows for the plasma properties to remain very similar regardless of the electrical properties of the sample. Recently mass transfer and associated reaction chemistry from a surface air plasma to a downstream aqueous sample has been studied, revealing a wealth of physicochemical events^10^. Reaction chemistry in the aqueous sample is found to be affected critically by the air gap between the surface plasma and the sample^10^,^16^. This suggests that the air gap may be used as a control to modulate aqueous chemistry, and in turn this modulates how the intended applications are achieved. Such process control is however challenged by the complexity of how short-lived reactive plasma species are transferred into the aqueous bulk of the downstream sample and how they may react with water molecules and long-lived plasma species to establish a dynamically evolving aqueous chemistry. For such control to be effective, it is also highly desirable to unravel main pathways that underpin aqueous chemistry. Little is known in literature of chemical pathways in the aqueous environment of the sample and indeed how they may be modulated by the air gap. The study presented here is motivated by the above knowledge gap.

### Experiment and simulation

We recently reported a system-level model for the interaction between surface air discharge and deionized water^10^. Brief descriptions of the model are presented in the Methods section, and for more detail please refer to ref. 10. The model was validated by comparing its predictions with experimental measurement, including O_3_ density in the gas phase, the pH value and densities of H_2_O_2_, O_3_, nitrate (HNO_3_ and NO_3_^−^) and nitrite (HNO_2_ and NO_2_^−^) in the PAW. For the air gap width of *L*_g_ = 1 cm, the numerical and experimental results were found to be in good agreement^10^. In this paper, the system-level model is used to study how aqueous chemistry may be modulated by varying the air gap width from *L*_g_ = 0.1 cm to 2 cm. This air gap range covers most application scenarios of the surface air discharge, for which heterogeneous mass transfer changes dramatically because of some short-lived species, such as HO_2_, having diffusion distances between 0.1 cm and 1 cm in air gap^10^. The dramatic changes in heterogeneous mass transfer, and consequently in the liquid chemistry is quantified by the system-level model, and is then validated by the experiments.

**Figure 1:**
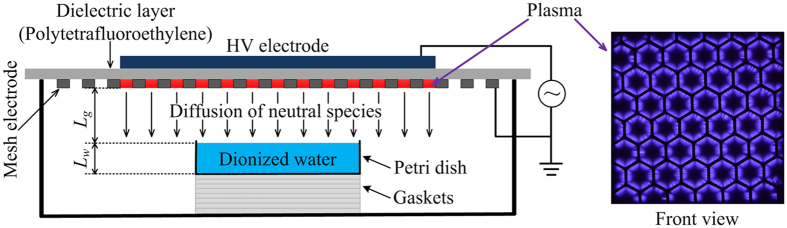
Schematic diagram of the experimental setup (the plasma image was taken by a camera with an exposure time of 0.2 s).

As shown in Fig. 1, the surface discharge structure consists of a plane high-voltage electrode, a liquid-facing grounded mesh electrode, and a dielectric sheet sandwiched between the two electrodes. A sinusoidal high voltage of *V*_*pp*_ = 11 kV and *f* = 10 kHz is applied to the high-voltage electrode with an averaged dissipated power density of 0.05 W/cm^2^. The surface plasma is confined in the mesh elements of the grounded electrode. As shown in Fig. 1, each mesh element has a hexagon shape, and the plasma has a good mesh-to-mesh homogeneity. The temperature of the mesh electrode measured with a thermocouple was found to remain roughly 300 K after 100 s of plasma treatment. The deionized water in a petri dish is placed underneath the plasma. The water depth *L*_w_ was constant to be 1 cm (except for the concentration measurement by electron spin spectroscopy), while the air gap between the plasma and the water surface is varied from *L*_*g*_ = 0.1 to 2 cm. The air gap width is adjusted by the changing thickness of gaskets under the petri dish. The diameter of the circular petri dish (3.5 cm) is much smaller than the width of the surface plasma, allowing for one-dimensional treatment in numerical simulation. The surface air plasma and the deionized water are well sealed by an organic glass box, which has a fixed chamber volume of ~493 cm^3^.

## Results and Discussion

### Long-lived species in the air gap and liquid regions

**Figure 2:**
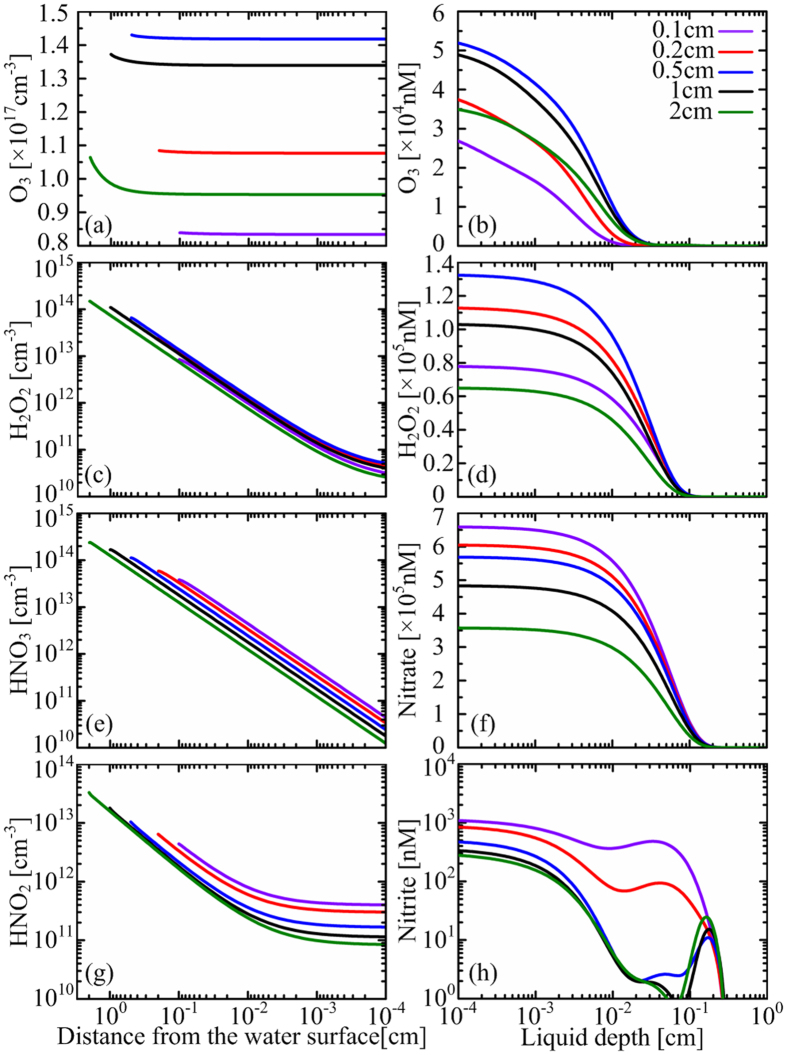
Spatial distributions of long-lived species in the air gap (left column) and the PAW (right column) at *t* = 100 s, for the air gap width variable from 0.1 to 2 cm.

From our system-level simulation, a large amount of O_3_, H_2_O_2_, N_2_O, N_2_O_5_, HNO_2_ and HNO_3_ can transfer from the gas phase into the deionized water. However, N_2_O does not react with other aqueous species and hence not discussed in this paper. By contrast, N_2_O_5_, HNO_2_ and HNO_3_ have strong reactions with the water molecules to form H^+^, NO_2_^−^ and NO_3_^−^. All the dissolved N_2_O_5_ transforms to H^+^ and NO_3_^−^ over a characteristic time of less than a microsecond. On the other hand, the dissolved HNO_2_ and HNO_3_ reach equilibrium with their hydrolyzed species H^+^, NO_2_^−^ and NO_3_^−^. Therefore, aqueous reactive species induced by the surface air discharge are mainly H^+^, O_3_, H_2_O_2_, nitrite and nitrate, consistent with those reported in literature^11^,^12^. The calculated density distributions of long-lived ROS and RNS in the air gap and in the PAW are shown in Fig. 2, for a plasma treatment time of *t* = 100 s. As the air gap increases from 0.1 to 2 cm, the densities of O_3_ and H_2_O_2_ in the PAW increase first and then decrease, peaking at L_g_ ~ 0.5 cm. By contrast the densities of nitrite and nitrate keep decreasing. It is suggested that O_3_ is the predominant antibacterial species in water treated by surface air plasmas^11^,^13^. Our simulation result may explain why typical sterilization efficiency has a trend of first rising and then falling as a function of the air gap width for a similar surface air discharge source^16^.

Ozone in the air gap is chemically stable, and its loss by dissolving in the water is limited due to the small Henry’s coefficient of ~0.23[ref. 17]. So if the treatment time is long enough, ozone accumulates in the air gap to achieve its equilibrium with the plasma and the water regions. This is the case for *L*_*g*_ ≤ 0.5 cm, in which the density profile is nearly flat across the whole air gap as shown in Fig. 2(a). The characteristic time for such equilibrium increases with the air gap width, and it becomes larger than 100 s when *L*_*g*_ > 0.5 cm, for which the density profiles drop in the vicinity of the plasma (see Fig. 2(a)). This is the reason why the O_3_ density in the air gap and consequently in the water decreases with increasing *L*_*g*_ when *L*_*g*_ > 0.5 cm. In the case of *L*_*g*_ < 0.5 cm, the increase of O_3_ density with the air gap width is due to the change of plasma chemistry. The O_3_ density in the air gap is around 10^17^ cm^−3^ for all the cases (see Fig. 2(a)), nearly 5% of its precursor O_2_, thus changing the background gas composition and consequently the plasma chemistry. This influence is more significant for shorter air gaps since the volume-averaged change is more pronounced.

The density of H_2_O_2_ in the air gap is lower than that of O_3_ by more than three orders of magnitude, but is higher in the PAW (see Fig. 2(b,d)), indicating that H_2_O_2_ has much larger dissolution rate than O_3_. This is also true for HNO_2_ and HNO_3_ since they also have large Henry’s coefficients as H_2_O_2_. So, the density profiles of gaseous H_2_O_2_, HNO_2_ and HNO_3_ drop dramatically near the water surface (Fig. 2(c,e,g)). It should be noted that the density of nitrite in the PAW is lower by comparison. This is because a large amount of nitrite is transformed to nitrate by reacting with O_3_ and NO_3_:


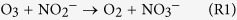



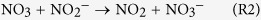


These are responsible for more than 90% of the loss of nitrite in the PAW. However, the penetration of the dissolved ozone lags behind nitrite, while NO_3_ is mainly exist in the topmost water layer of *L*_*w*_ < 100 μm (will discussed below), leading to a local density maximum of nitrite at the front part of the profiles (see Fig. 2(h)).

**Figure 3:**
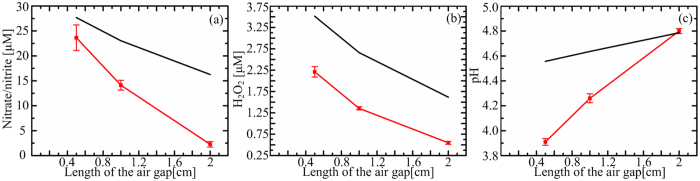
Comparison between the numerical (black fold line) and experimental (red fold line) results for (**a**) the nitrite/nitrate densities, (**b**) the hydrogen peroxide densities and (**c**) the pH values.

The pH value, representing the density of H^+^, and the volume-averaged densities of H_2_O_2_ and nitrate/nitrite in the PAW were measured for *L*_*g*_ = 0.5, 1 and 2 cm, as shown in Fig. 3. By comparison, the numerical and experimental results have similar variation trends. Quantitatively, the numerical results are slightly higher, 1.6 ~ 2.9 fold higher for H_2_O_2_, 1.2 ~ 7.2 fold higher for nitrite/nitrate, and for the pH value the maximum difference is 0.6. This confirms that our model is capable of capturing the main physicochemical processes.

### Short-lived species in the air gap and liquid regions

**Figure 4:**
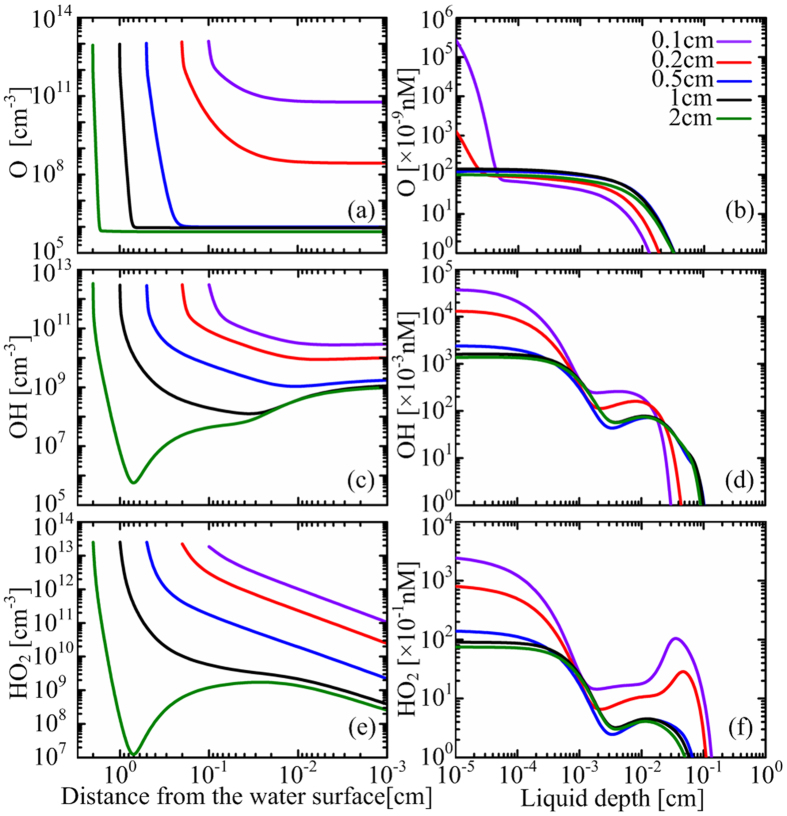
Spatial distributions of short-lived ROS in the air gap (left column) and the water (right column) at t = 100 s, for the air gap width variable from 0.1 to 2 cm.

Among short-lived ROS, O, OH and HO_2_ are widely thought to be crucial for various applications^1^. They are known to have short lifetimes due to their highly reactivity, so it is unclear whether they can play an important role when the target to be treated is immersed in water. The spatial distributions of such three short-lived ROS in the air gap and in the PAW are shown in Fig. 4.

As shown in Fig. 4(a,b), the density of atomic oxygen at the gas-water interface remains about the same for *L*_*g*_ ≥ 0.5 cm. When *L*_*g*_ < 0.5 cm, however, it increases by more than one order of magnitude with the decreasing *L*_*g*_. This indicates that a non-negligible amount of the atomic oxygen from the surface air plasma can diffuse across the air gap and dissolve into the water. According to the Einstein-Smulochowski equation, *i.e.* 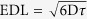 with *D* being the diffusion coefficient and τ the lifetime of a species, the effective diffusion distance in lifetime (EDL) of atomic oxygen in the air gap was estimated to be less than 0.02 cm^10^. It is consistent with this study since its density decreases by ~6 fold at 0.02 cm from the plasma (see Fig. 4(a)). However, the critical gap width within which the heterogeneous mass transfer has marked effect on the density of aqueous atomic oxygen is larger by more than one order of magnitude. In the cases of *L*_*g*_ ≤ 0.2 cm, the density profiles of atomic oxygen in the PAW have a sharp fall in the surface layer of water with a depth *L*_*w*_ < 1 μm (see Fig. 4(b)), indicating a similar short penetration depth of the dissolved atomic oxygen. In the deeper region of the PAW where the dissolved atomic oxygen cannot reach, the aqueous atomic oxygen has parabolic density profiles for all air gaps studied, which should be attributed to liquid chemistry.

The density of aqueous OH also increases significantly with decreasing *L*_*g*_ when *L*_*g*_ < 0.5 cm (see Fig. 4(d)), but it is not due to the mass transfer from gas phase to liquid phase. The direction of heterogeneous mass transfer of OH is reversed for all the air gap widths (see the chemical profile below). This is why the density profiles of OH in the air gap have a concave shape for *L*_*g*_ ≥ 0.5 cm (Fig. 4(c)). So, the evolution of density profiles of aqueous OH is mainly due to the change in liquid chemistry, largely by the reaction as follows:


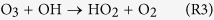


The aqueous O_3_ density decreases sharply at *L*_*w*_ > 10 μm(see Fig. 2), leading to a two-step OH density shape with an interface at *L*_*w*_ = 10 ~ 30 μm.

The heterogeneous mass transfer of HO_2_ has a constant direction from the gas phase to the liquid phase, seen in the downward trends of the HO_2_ density around the gas-liquid interface (Fig. 4(e,f)). However, the plasma-generated HO_2_ is not capable of traversing the air gap of *L*_*g*_ > 1 cm, as reflected in the concave shape of the density profile for *L*_*g*_ = 2 cm (see Fig. 4(e)). The heterogeneous mass transfer flux of HO_2_ is supplied mainly by diffusion from the plasma region, dominant for *L*_*g*_ < 0.5 cm, and by the gaseous reaction of O_3_ + OH → HO_2_ + O_2_ which dominates for *L*_*g*_ ≥ 0.5 cm.

**Figure 5:**
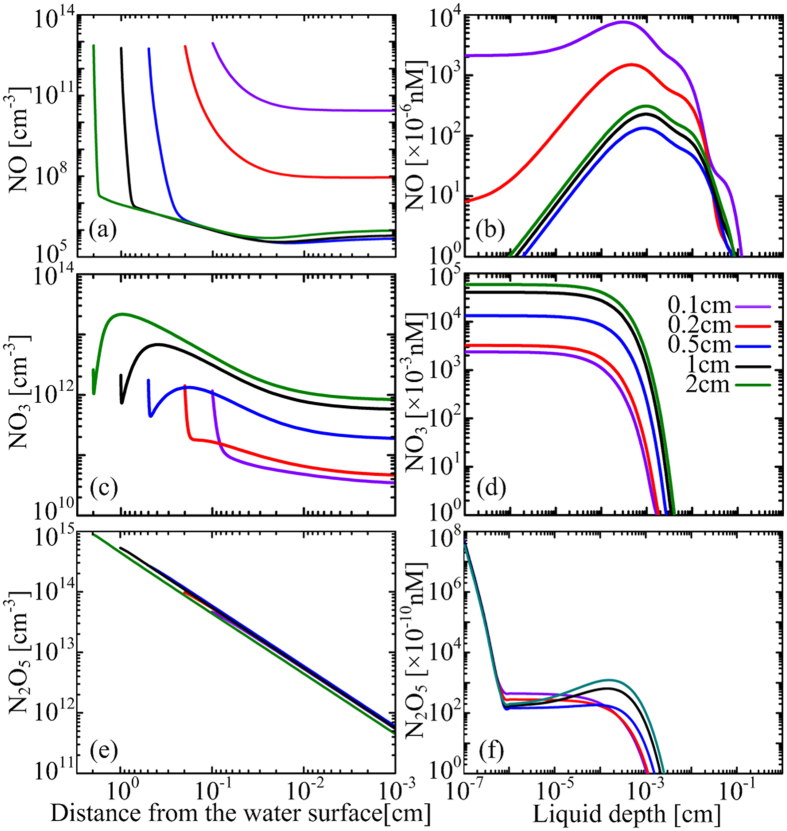
Spatial distributions of short-lived RNS in the air gap (left column) and the water (right column) at *t* = 100 s, for the air gap width variable from 0.1 to 2 cm.

Density distributions of NO, NO_3_ and N_2_O_5_ in the air gap and the water at *t* = 100 s are plotted in Fig. 5. NO is a well known signaling molecule in biological system^18^, where NO_3_ is important for the generation of short-lived aqueous species (discussed below). N_2_O_5_ is very stable in the gas phase, but it becomes highly reactive in the PAW and hence treated as a short-lived RNS here.

Similar to O and OH, the density of NO across the gas-water interface remains about the same for *L*_*g*_ ≥ 0.5 cm (see Fig. 5(a)). However, it increases sharply with decreasing *L*_*g*_ when *L*_*g*_ < 0.5 cm. The heterogeneous mass transfer of NO is also from the liquid phase to the gas phase as it is the case for OH, so the aqueous NO is totally generated by liquid chemistry. The main reactions are as follows:


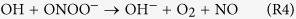



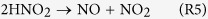


NO_3_ is chemical active in both gas and liquid phases^19^,^20^. Yet, it has not been reported to play an important role in plasma applications. The gaseous NO_3_ density at the gas-liquid interface increases from 3.2 × 10^10^ to 8.3 × 10^11^ cm^−3^ for *L*_*g*_ = 0.5–2 cm (see Fig. 5(c)), but they are not diffused from the plasma even for the case of *L*_*g*_ = 0.1 cm due to its high reactivity in the air gap (see the sharp fall of NO_3_ at the left side of each density curve in Fig. 5(c)). Our numerical results suggest that heterogeneous mass transfer supplies more than 97% of the NO_3_ in the PAW, and in gas phase those NO_3_ are almost generated by the following reactions:


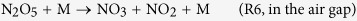



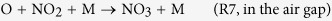



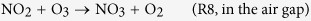


R6 is important for all the gap widths, but R7 and R8 are important only for *L*_*g*_ < 0.5 cm.

The densities of gaseous N_2_O_5_ decrease linearly towards the gas-water interface (see Fig. 5(e)), indicating that this species is mainly consumed by the dissolution as it is the case for HNO_3_ (see Fig. 2(e)). However, it strongly reacts with water molecules and hence its density decreases by more than two orders of magnitude when penetrating just 1 nm in the water (see Fig. 5(f)). Similar to the atomic oxygen, N_2_O_5_ exists in the deeper region of the PAW where the dissolved N_2_O_5_ cannot reach, mainly due to the liquid reaction as given by


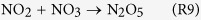


**Figure 6:**
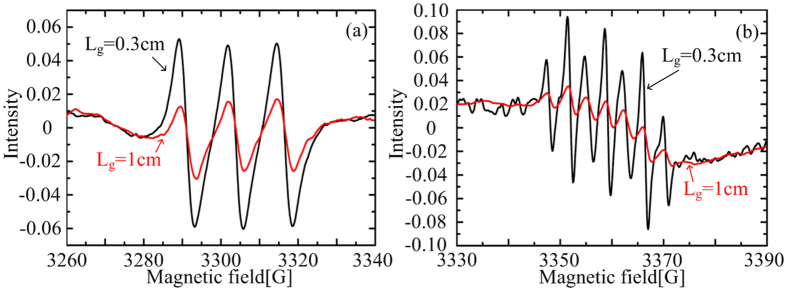
ESR spectra of the PAW for the air gap widths of 0.3 cm and 1 cm (Water depth = 0.1 cm; Treatment time = 5 min).

The calculated results suggest that free radicals such as OH exist in the deionized water. This may appear counter intuitive because their lifetimes are very short while the plasma is atmost 2 cm away from the water. In order to prove the existence of free radicals in the PAW, OH and NO were detected using electron spin resonance (ESR). Spin trapping reagents were added into the water before the plasma treatment, reacting specifically with short-lived radicals to form long-lived spin adducts, which then accumulate to a sufficiently high level to be detected^21^. So, the ESR results represent the relative values of the volume-averaged densities of free radicals in the PAW. Since the densities of aqueous OH and NO are very low (Figs 4(d) and 5(b)), for this measurement the water depth is reduced to 0.1 cm, and the treatment time is increased to 5 min. The reduction of water depth leads to the increase of volume-averaged densities, because free radicals mainly exist in the surface layer of water (see Figs 4(d) and 5(b)). Also, the increase of the plasma treatment time allows the spin adducts to accumulate allowing for easy ESR measurement. In this case, the spin adduct of NO was detected for an air gap width of 0.3 cm and 1 cm, as shown in Fig. 6(a). Its density increases as the decrease of the air gap width, in accordance with the simulation results of NO (see Fig. 4(d)). This confirms that the surface air discharge can remotely induce the generation of short-lived species in the downstream water sample, thus supporting our hypothesis and also our numerical model. It should be noted that direct evidence for the existence of OH radicals in the water is still lacking. Fig. 6b shows clear disruption to the ESR spectrum of OH radicals, mostly probably by other free radicals, which results in a distorted spectrum of seven peaks instead of the classic four-peak ESR spectrum of OH radicals. This is also observed in an O_3__-_rich solution similar to our case^22^.

### Chemical pathways in the PAW

From Figs 2, 3, 4, 5, 6, it can be concluded that the aqueous reactive species are induced by both the heterogeneous mass transfer and the liquid chemistry and this dual influence is significantly influenced by the air gap width. Also, two distinct scenarios appear to exist, of which the density profiles are considerably different: Scenario 1 (S1 for abbreviation) for *L*_*g*_ < 0.5 cm and scenario 2 (S2) for *L*_*g*_ > 0.5 cm. The scenarios are also true for the chemical pathways in the PAW, as will be discussed below.

**Figure 7:**
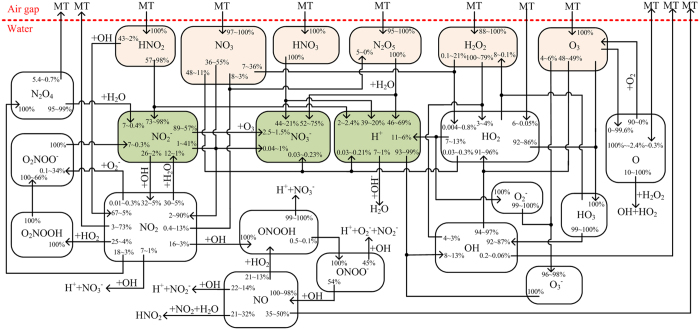
Chemical profile of aqueous reactive species induced by the surface air plasmas for the air gap width from 0.1 to 2 cm.

Taken the results and their discussions together, we summarize the network of numerous mutually coupled production mechanisms of aqueous plasma species in Fig. 7. Here, MT represents the heterogeneous mass transfer, and each percentage number indicates the volume-averaged relative contribution of a specific physicochemical process to the production/loss of a species at *t* = 100 s. The relative contribution of heterogeneous mass transfer is calculated by dividing the net density flux of a species at the gas-liquid interface by the water depth, i.e. flux/*L*_*w*_. It should be noted that the production rate might be much different to the loss rate even when their relative contributions are the same. This is because the species themselves may not be in equilibrium. Taking HNO_2_ for example, the gas-liquid mass transfer contributes ~100% on its production, while it losses mainly by hydrolysis which contributes ~57% when *L*_*g*_ = 0.1 cm and ~98% when *L*_*g*_ = 2 cm. The contribution of MT on production of atomic oxygen is of particular interest, it is ~100% for *L*_*g*_ = 0.1 cm, but decreases to about −2.4% when *L*_*g*_ = 0.5 cm, i.e. the direction of MT reverses, and then increases to 0.3% when *L*_*g*_ = 2 cm as the direction of MT reverses again. The big change of MT contribution on the production of atomic oxygen results in a significant change in its liquid reaction. When *L*_*g*_ = 0.1 cm, the dissolved atomic oxygen is mainly consumed by reacting with the dissolved molecular oxygen as given by


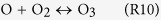


However when the dissolution of atomic oxygen is limited (*L*_*g*_ ≥ 0.5 cm), this reaction becomes instead the main production pathway of atomic oxygen in the PAW (see Fig. 7).

The heterogeneous mass transfer is dominated by gaseous species with high densities, including HNO_2_, HNO_3_, H_2_O_2_, O_3_, N_2_O_5_ and N_2_O. N_2_O is chemically stable and hence not shown in Fig. 7. The hydrolysis of HNO_2_ and HNO_3_ is very strong, with a total reaction rate of ~10^15^ cm^−3^s^−1^, indicating that in a topmost water layer as thin as ~1 μm most of them transform to H^+^, NO_2_^−^ and NO_3_^−^. Also, N_2_O_5_ is an important precursor for H^+^ and NO_3_^−^. It reacts strongly with water molecules and hence its penetration depth is less than 1 nm as shown in Fig. 5(f). So, a surface water layer exists with *L*_*w*_ < 1μm, within which chemical reactions are mainly induced by the dissolved HNO_2_, HNO_3_ and N_2_O_5_, much different to the bulk water region. By comparison, the heterogeneous mass transfer of other species, including NO_3_, HO_2_, NO, OH and N_2_O_4_, is much less significant, and for NO, OH, NO_2_ and N_2_O_4_ the mass transfer direction reverses (see Fig. 7).

**Figure 8:**
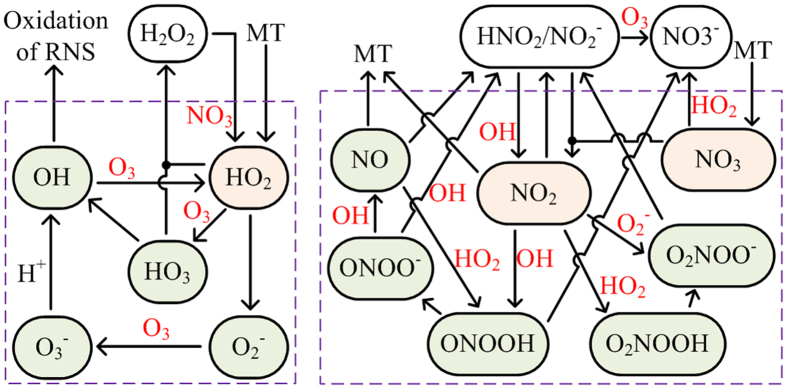
Chemical pathways for the short-lived ROS and RNS in the PAW.

Liquid chemistry is the direct mechanism for production of most reactive species in the PAW as shown in Fig. 7. This is why some short-lived species exist in the PAW regardless whether they can be supplied from the gas phase. The chemical pathways among the aqueous species are complex. In Fig. 8(a,b), we present a network of simplified chemical pathways for short-lived ROS and RNS. In order to distinguish between short-lived species and long-lived species, short-lived ones are shown in the dashed boxes. Some short-lived species are not included because their densities are very low, such as the atomic oxygen, or their biological effects are not known to be important. For short-lived ROS, there are two main reaction cycles among them: (1) OH → HO_2_ → HO_3_ → OH, and (2) is OH → HO_2_ → O_2_^−^ → O_3_^−^ → OH (see Fig. 8(a)). The first cycle has a reaction rate between 7.2 × 10^13^ and 3.4 × 10^14^ cm^−3^s^−1^, while the second is between 1.1 × 10^13^ and 2.7 × 10^13^ cm^−3^s^−1^, both decreasing with increasing gas gap width. It is interesting that O_3_ acts as an intermediate for both reaction cycles, in which it loses an O atom to become O_2_. So, the reaction cycles lead to no net production/reduction of the short-lived ROS as a whole but to a reduction of O_3_ with a high reaction rate.

Considering the short-lived ROS as a whole (in the dashed box of Fig. 8(a)), there are two main production pathways: one is the dissolution of HO_2_ from the gas phase, and the other is the generation of HO_2_ by the reaction between the dissolved NO_3_ and H_2_O_2_ as follows:


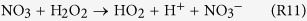


The first one dominates in S1, while the second dominates in S2. HO_2_ is the original short-lived ROS in the PAW, and its production rate by both pathways is between 7.0 × 10^11^ and 2.1 × 10^13^ cm^−3^s^−1^, smaller than that of the internal reaction cycles of short-lived ROS by more than one order of magnitude. This is why they contribute less than 10% of the HO_2_ production (see Fig. 7).

For the loss of short-lived ROS as a whole, there are also two main pathways: one is the reaction between HO_2_ and HO_3_ to form H_2_O_2_, of which the rate decreases from 1.8 × 10^12^ to 1.2 × 10^10^ cm^−3^s^−1^ with increasing air gap. The other is the reduction of OH, O_2_^−^ and HO_2_ by various RNS as shown in Fig. 8(b). The total reduction rate increases from 6.4 × 10^6^ cm^−3^s^−1^ to 1.8 × 10^11^ cm^−3^s^−1^ with *L*_*g*_, indicating that the first pathway (HO_2_ + HO_3_ → H_2_O_2_ + 1.5 O_2_) dominates in S1, while the other dominates in S2.

For the short-lived RNS in the PAW, NO_2_ is the precursor for the other species except for NO_3_. NO_2_ is mainly produced by the advanced oxidation of nitrite in S1 as given by:


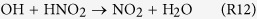



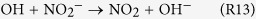


Since the densities of nitrite (HNO_2_/NO_2_^−^) and OH decrease with increasing air gap width (see Fig. 4(h) and 5(d)), the production rate of this pathway decreases accordingly. However, the dissolved NO_3_ has its density increases by more than one order of magnitude with the air gap width from 0.1 cm to 2 cm (see Fig. 5(d)), and hence a new pathway dominates the production of NO_2_ in S2, as given by


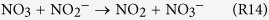


As shown in Fig. 7, R14 contributes ~90% of the production of NO_2_ when *L*_*g*_ = 2 cm.

It is interesting that short-lived ROS such as OH play as an essential intermediate for the transformation among the RNS (see Fig. 8(b)), which leads to the production of various kinds of short-lived RNS, such as ONOOH known to have strong biological effects^23^. In turn, it enables a main pathway for the reduction of ROS as discussed above.

Regarding the loss of short-lived RNS as a whole, there are two main pathways: one is the transformation to nitrite and to nitrate, mostly by:


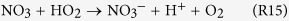



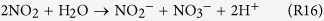



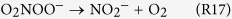



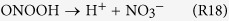



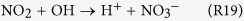


The total reaction rate of R15 ~ R19 is from 2.6 × 10^12^ cm^−3^s^−1^ to 1.5 × 10^11^ cm^−3^s^−1^, decreasing as the increase of the air gap width, and R17 dominates in S2 since it has a rate more than half of the total.

The other pathway is the mass transfer of NO and NO_2_ from the liquid phase to the gas phase, which has a total flux diving the liquid depth (Flux/*L*_*w*_) from 1.3 × 10^11^ cm^−3^s^−1^ to 1.1 × 10^12^ cm^−3^s^−1^ at the gas-liquid interface, increasing with the air gap width. As a result, the liquid chemistry dominates the reduction of short-lived RNS in S1, while the heterogeneous mass transfer dominates in S2. In biological solutions where the organic species have intensive reactions with the RNS, it is likely that short-lived RNS are consumed by liquid chemistry, and the heterogeneous mass transfer is compromised.

From the discussions above, the main pathways of heterogenous mass transfer and liquid chemistry are described and analyzed. Although the discharge power density is fixed at 0.05 W/cm^2^, the pathways should remain valid for a large range of input power since the density ratios of reactive species change little. However, it should be noted that the surface discharge may transfer to the ozone-poisoning mode when the discharge power density is larger than ~0.2 W/cm^2^ for our experimental setup, in which the ozone density may be reduced by several orders of magnitude^24^,^25^. In such a situation, the gas composition of reactive species is dramatically changed and consequently it affects the heterogenous mass transfer and liquid-phase chemistry^11^,^25^. The influence of the discharge power on the interaction between the surface air discharge and deionized water will be reported in future.

## Conclusion

In conclusion, the interaction between a surface air discharge and deionized water has been studied with a system-level model and selected experimental measurements. Long-lived aqueous plasma species are H^+^, nitrate, nitrite, H_2_O_2_ and O_3_. In addition, various short-lived ROS and RNS are also induced in water, regardless whether these species are supplied from the gas phase. Our results show that the aqueous reactive species are controlled by heterogeneous mass transfer and/or liquid chemistry. Short-lived ROS and RNS in water are strongly coupled by liquid chemistry: NO_3_ is an important precursor for HO_2_ and successively for the other short-lived ROS. On the other hand, OH, O_2_^−^ and HO_2_ play a crucial role for the production of various kinds of RNS. Moreover, several internal chemical chains exist among the short-lived ROS or RNS with high reaction rates. For example, the chain of OH → HO_2_ → HO_3_ → OH has a reaction rate between 7.2 × 10^13^ and 3.4 × 10^14^ cm^−3^s^−1^, but leads to no net production/reduction of the short-lived ROS as a whole. On the other hand, O_3_ is reduced since it acts as an intermediate of this chain. The heterogeneous mass transfer is strongly dependent on the air gap width, mainly because the diffusion distances of the short-lived species are typically between 0.1 and 1 cm. This further influences the liquid chemistry since the dissolved amount of the short-lived species changes in accordingly.

The influence of heterogeneous mass transfer and aqueous plasma chemistry appears to vary significantly in two distinct scenarios. In the first scenario with *L*_*g*_ < 0.5 cm, the concentrations of the long-lived aqueous ROS, i.e. H_2_O_2_ and O_3_, increase with *L*_*g*_, and the short-lived ROS, such as O and HO_2_, can diffuse across the air gap and then dissolve into water. The dissolved HO_2_ is the main precursor for the other short-lived ROS, and as a whole the short-lived ROS is mainly reduced by the reaction between HO_2_ and HO_3_ to form H_2_O_2_. Regarding the aqueous short-lived RNS as a whole, it is mainly produced by the advanced oxidation of nitrite to form NO_2_ and successively the other species (except for NO_3_ which is from the heterogeneous mass transfer), and reduced mainly by transforming to nitrite and nitrate.

In the second scenario of *L*_*g*_ > 0.5 cm, on the other hand, the concentrations of aqueous H_2_O_2_ and O_3_ decrease with increasing *L*_*g*_. HO_2_ is mainly produced the liquid reaction between NO_3_ and H_2_O_2_, and as a whole the short-lived ROS is reduced by reacting with RNS. Regarding the aqueous short-lived RNS as a whole, it is mainly produced by the heterogeneous mass transfer of NO_3_, and reduced mainly by the mass transfer of NO_2_ from the liquid phase to the gas phase.

## Methods

The system-level model consists of three modules for the plasma generation region, the air gap region, and the deionized water region, respectively, and all three modules are calculated together simultaneously. A zero-dimensional module is used for the surface plasma in humid air, of which 53 species and 624 chemical reactions are incorporated. The densities of gaseous plasma species are obtained by calculating the production/loss rates of the corresponding chemical reactions, the particle fluxes between the plasma region and the air gap region, and the dissipated power density for accelerating the charged species which equals to 0.05 W/cm^2^. Only neutral species are considered in the air gap region, so the amount of the species and the corresponding reactions are reduced to 21 and 63, respectively. The diffusion and chemical reactions of the neutral species in the air gap, as well as their particle fluxes onto the plasma-gas interface and the gas-liquid interface, are calculated by the one-dimensional module of the air gap region. Henry’s law was used to describe the density relationship of species on both sides of the gas-liquid interface. Although only neutral species can dissolve into the water from the air gap region, some of them (mainly HNO_2_ and HNO_3_) strongly hydrolyze to form numerous ionic species (mainly H^+^, NO_2_^−^ and NO_3_^−^) in the water, and hence a drift-diffusion equation and Possion’s equation are used to describe the behavior of species in the PAW. The module for the deionized water is also one-dimensional, which incorporates 33 species and 109 chemical reactions, including 21 reversible reactions. All the species considered in the system-level model are listed in Table 1. It is noted that several assumptions in the model were made by carefully consideration. For example, the diffusion of charged species from the plasma into the air gap is neglected, because their diffusion distances in lifetimes are less than 100 μm^10^ and therefore such diffusion has little effect on the remote liquid region which is focused in this paper. The temperature is fixed to be 300 K for both the gas and liquid phases, which is very close to the measured values even for the plasma region.

For experiments used in this study, a pH probe (Sartorius, PB-10) was used, and the concentrations of aqueous H_2_O_2_ and nitrate/nitrite were measured by using a microplate reader (Thermo Scientific Varioskan® Flash Reader). The Amplex® Red reagent was added into the water right after the SMD treatment, and it reacted with H_2_O_2_ in a 1:1 stoichiometry to produce the red-fluorescent oxidation product, which was excited at λ = 550 nm and emitted at λ = 595 nm. Similarly, the Griess reagent was added into the water to detect the nitrate/nitrite, and the absorbance was measured at λ = 550 nm. For the measurement of OH and NO, the spin trapping reagents were added into the water before the plasma treatments, and their spin adducts were measured by an X-band ESR (BrukerBioSpin GmbH, EMX) immediately after the plasma treatments. A spin trapping reagent, 5,5-dimethyl-1-pyrrolineN-oxide (DMPO) was used to trap OH, and the spin adduct of DMPO·/OH has a special ESR spectra with a peak intensity ratio of 1:2:2:1. Also, the DETC-Fe^2+^ complex was used to trap NO, and the spin adduct, (DETC)_2_-Fe^2+^ −NO, has a special ESR spectra with a peak intensity ratio of 1:1:1. All the measurements above were repeated three times.

## Additional Information

**How to cite this article**: Liu, D. X. *et al*. Aqueous reactive species induced by a surface air discharge: Heterogeneous mass transfer and liquid chemistry pathways. *Sci. Rep.* **6**, 23737; doi: 10.1038/srep23737 (2016).

## Figures and Tables

**Table 1:** Species considered in the model.

	Cations	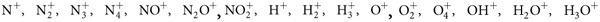
Plasma region	Anions	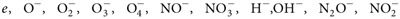
	Neutrals	N(^2^*D*), N_2_(*A*^3^Σ), N2(*B*^3^Π), H, N, H_2_, N_2_, H_2_O, O(^1^*D*), O, O_2_(*a*^1^Δ), O_3_, OH, HO_2_, H_2_O_2_, O_2_, NO, NO_2_, NO_3_, N_2_O_3_, N_2_O_4_, N_2_O_5_, HN*O*_2_, HNO_3_, N_2_O, HNO
Air gap region	NO, N_2_O, NO_2_, NO_3_, N_2_O_3_, N_2_O_4_, N_2_O_5_, HNO, HNO_2_, HNO_3_, N, N_2_, O_2_, O, *O*_2_(*a*^1^Δ), O_3_, OH, H_2_O_2_, HO_2_, H_2_, H_2_O	
Liquid region	O, O_3_, OH, HO_2_, HO_3_, H_2_O_2_, N_2_, O_2_, H_2_O, H, H_2_, N_2_O_3_, NO, NO_2_, NO_3_, N_2_O_4_, N_2_O_5_, HNO_2_, H ^+^ 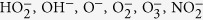 , 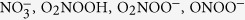 ,